# Association Analysis of NLRP3 Inflammation-Related Gene Promotor Methylation as Well as Mediating Effects on T2DM and Vascular Complications in a Southern Han Chinese Population

**DOI:** 10.3389/fendo.2018.00709

**Published:** 2018-11-29

**Authors:** Zixing Zhou, Lijun Wang, Zihao Wen, Shaoling Zheng, Xiaohong Ye, Dandan Liu, Jing Wu, Xiaoqian Zou, Yumei Liu, Yao Wang, Shirui Dong, Xiuxia Huang, Xiuben Du, Kehui Zhu, Xiaojing Chen, Shiqi Huang, Chengli Zeng, Yajing Han, Baohuan Zhang, Lihong Nie, Guang Yang, Chunxia Jing

**Affiliations:** ^1^Department of Epidemiology, School of Medicine, Jinan University, Guangzhou, China; ^2^Department of Nutriology, School of Medicine, Jinan University, Guangzhou, China; ^3^Department of Endocrine, The First Affiliated Hospital of Jinan University, Guangzhou, China; ^4^Department of Pathogen Biology, School of Medicine, Jinan University, Guangzhou, China; ^5^Guangzhou Key Laboratory of Environmental Exposure and Health, Guangdong Key Laboratory of Environmental Pollution and Health, Jinan University, Guangzhou, China

**Keywords:** T2DM, vascular complication, NLRP3, AIM2, ASC, promoter methylation

## Abstract

**Objective:** To explore the association between the methylation levels in the promoter regions of the NLRP3, AIM2, and ASC genes and T2DM and its vascular complications in a Southern Han Chinese population and further analyze their interaction and mediating effects with environmental factors in T2DM.

**Methods:** A case-control study was used to determine the association between population characteristics, the methylation level in the promoter region of the NLRP3, AIM2, and ASC genes and T2DM and vascular complications. A mediating effect among genes-environment-T2DM and the interaction of gene-gene or gene-environment factors was explored.

**Results:** In the logistic regression model with adjusted covariants, healthy people with lower total methylation levels in the AIM2 promoter region exhibited a 2.29-fold [OR: 2.29 (1.28~6.66), *P* = 0.011] increased risk of developing T2DM compared with higher-methylation individuals. T2DM patients without any vascular complications who had lower methylation levels (<methylation median) in NLRP3 CpG2 and AIM2 total methylation had 6.45 (OR: 6.45, 95% CI: 1.05~39.78, *P* = 0.011) and 9.48 (OR: 9.48, 95% CI: 1.14~79.00, *P* = 0.038) times higher risks, respectively, of developing diabetic microvascular complications than T2DM patients with higher methylation. Similar associations were also found between the lower total methylation of the NLRP3 and AIM2 promoter regions and macrovascular complication risk (NLRP3 OR: 36.03, 95% CI: 3.11~417.06, *P* = 0.004; AIM2 OR: 30.90, 95% CI: 2.59~368.49, *P* = 0.007). Lower NLRP3 promoter total methylation was related to a 17.78-fold increased risk of micro-macrovascular complications (OR: 17.78, 95% CI: 2.04~155.28, *P* = 0.009). Lower ASC CpG1 or CpG3 methylation levels had significant partial mediating effects on T2DM vascular complications caused by higher age (ASC CpG1 explained approximately 52.8% or 32.9% of the mediating effect of age on macrovascular or macro-microvascular complications; ASC CpG3 explained approximately 38.9% of the mediating effect of age on macrovascular complications). No gene-gene or gene-environment interaction was identified in T2DM.

**Conclusion:** Lower levels of AIM2 promoter total methylation might increase the risk of T2DM. NLRP3, AIM2, and ASC promoter total methylation or some CpG methylation loss might increase the risk of T2DM vascular complications, which merits further study to support the robustness of these findings.

## Introduction

The International Diabetes Federation (IDF) reported that 1 in 11 adults had diabetes (415 million) in 2015, which is predicted to rise to 1 in 10 adults (642 million) by 2040 globally. One in 7 births is affected by gestational diabetes, and every 6 seconds a person dies from diabetes in the world. More than 95% of these adult patients have type 2 diabetes mellitus (T2DM) (1). The number of Chinese diabetes patients is the highest in the world (109.6 million in 2015), resulting in a heavy economic burden (2).

T2DM is characterized by damage to insulin secretion and sensitivity, resulting in hyperglycemia, which promotes T2DM micro-macrovascular complications, such as cerebral infarction (DCI) and diabetic retinopathy (DR) (3). It has been reported that a series of inflammatory responses are closely related to the pathogenesis of T2DM and its vascular complications (3–5). The NLRP3 inflammasome, belonging to the nucleotide-binding oligomerization domain (NOD)–like receptor (NLR) family, is the most fully characterized of the inflammasomes and contains the adaptor protein apoptosis-associated specklike protein (ASC), the proinflammatory caspase-1 and NLRP3, which can form caspase-1-activating inflammasomes (5). Absent in melanoma 2 (AIM2), an HIN-200 family member, can also drive assembly of a caspase-1-activating inflammasome (6). Various host-derived danger signals such as indicators of cell damage (“danger-associated molecular patterns” or DAMPs) and environmental irritants can activate the NLRP3 inflammasome, resulting in IL-1β and IL-18 production (7, 8). Hence, IL-1β and IL-18 maturation and secretion can promote inflammatory immune cell infiltration, contribute to islet β cell death and dysfunction and result in T2DM (5).

Several studies have demonstrated that epigenetic factors, such as DNA methylation and histone modifications, might influence the pathogenesis of T2DM (9, 10). It was proposed that DNA methylation could regulate gene expression in pancreatic islets of T2DM patients and subsequently contribute to impaired insulin secretion (10). DNA methylation of gene promoter regions is known to play an important role in the transcriptional regulation of genes (11). The variation in DNA methylation within the INS gene promoter is able to modify INS transcription and expression in pancreatic β cells and islets in diabetic patients (12, 13). Thus, we hypothesized that a DNA promoter methylated variant of the NLRP3, AIM2 and ASC genes might induce a large secretion of IL-1β and IL-18, contribute to islet β cell death and dysfunction and impaired insulin secretion, and increase the risk of T2DM and vascular complications in a Han Chinese population.

## Materials and methods

### Study subjects

In this case-control study, 93 simple randomly chosen T2DM cases and 93 simple randomly chosen healthy controls without T2DM family history were collected from the Overseas Chinese Hospital in Guangzhou from September 2013 to January 2015. All participants were Chinese Han residents who gave informed consent. T2DM patients were diagnosed by the 2003 American Diabetes Association criteria (14). Patients with T1DM and abnormal glucose tolerance tests were excluded from the study. The committee for the Ethics of Human Research of the School of Medicine in Jinan University approved the study protocol. Among T2DM patients, 28 cases were diagnosed with T2DM without any complication, whereas 15, 25 and 25 patients had microvascular complications, macrovascular complications, and micro-macrovascular complications, respectively. T2DM without any complication means without both microvascular and macrovascular complications. Microvascular complications include diabetic retinopathy (DR), diabetic nephropathy (DN) and diabetic neuropathy (DN), whereas macrovascular complications are coronary artery disease (CAD) leading to angina or myocardial infarction, peripheral artery disease (PAD) contributing to stroke and diabetic encephalopathy. Micro-macrovascular complications comprise both microvascular and macrovascular complications (15–17).

### DNA extraction and bisulfite conversion

All the tests applied the standard procedures recommended by the manufacturers. Genomic DNA was extracted from peripheral whole blood samples using a QIAamp Blood DNA Mini Kit (Qiagen, Hilden, Germany). Treatment of DNA with bisulfite results in the conversion of unmethylated cytosines to uracils, while methylated cytosines remain unaltered. Briefly, 5.5 μL of 3 M NaOH was added to 1 μg DNA (50 μL volume) and incubated at 42°C for 20 min. Following the incubation step, 520 μL of 3.6 M NaHSO3 (pH 5.0) and 30 μL of 10 mM hydroquinone were added to each sample (fresh and avoiding exposure to light). All reagents were gently mixed, centrifuged, covered with mineral oil, and then incubated at 53°C for 16 h. The bisulfite-converted DNA was purified by a Wizard DNA Clean-Up System (Promega, USA). Finally, DNA was eluted with 54 μL of DDW at 80°C, and 6 μL of 3 M NaOH was added to each sample, followed by incubation at 42°C for 15 min. Following the incubation, DNA was precipitated using 6 μL of 3 M sodium acetate and 30 μL of cold 100% ethanol at −80°C for 3 h. Each sample was centrifuged for 30 min at 12,000 g, and the supernatant liquid was discarded. The bisulfite-converted DNA was washed in 70% ethanol, dried and finally resuspended in a total volume of 10 μL.

### PCR amplification and BSP analysis of gene promoter methylation

The modified DNA was subsequently used as the template for PCR amplification using a PCR kit (TaKara EpiTaq™ HS for bisulfite-treated DNA, Japan). The cycle conditions were as follows: Initial denaturation at 98°C for 3 min; 40 cycles consisting of denaturation at 98°C for 10 s, annealing at 53°C for 30 s and extension at 72°C for 30 s; and final extension at 72°C for 10 min. The PCR product was sequenced by the Guangzhou Aiji Biotechnology Institute (Guangzhou, China). The methylation level was calculated as C/(C+T), total methylation = (CpG1+CpG2+…+CpGn)/n. The promoter BSP primer was designed online (http://www.urogene.org/methprimer/) (18). The sequences of the primers are shown in Table S1.

### Statistical analyses

The statistical analyses were done with GraphPad Prism 5.0 (GraphPad Software, US) and the Statistical Product and Service Solutions software 24.0 (SPSS 24.0) (IBM Corp, Armonk, NY, USA).

We performed Shapiro-Wilk test to identify the normality of the including clinical characteristics and the methylation data in each group (Table S4). The clinical characteristics of the study population were described as the mean ± S.D. or number (%) and the methylation data were expressed as the median and range. The *T*-test and χ^2^ test were used to compare clinical parameters between healthy controls and T2DM cases. For the continuous variables, the *t*-test and one-way analysis of variance (ANOVA) were used for those with normal distribution, otherwise, the Mann-Whitney test and Kruskal-Wallis test would be performed. The χ^2^ test was used to analyze the binary variables in each group. In T2DM subgroups multiple comparisons, the Dunnett method was used in ANOVA, and Dunn-Bonferroni method was used in Kruskal-Wallis test.

A logistic regression model was used to analyze the association between NLRP3, AIM2 and ASC promoter methylation and T2DM or its vascular complications after adjusting for covariates, including age, gender, and BMI, which were significantly different in baseline characteristics. The methylation level in the regression model was divided into a binary variable (≥ and <methylation median) (Table S2). All of the AIM2 CpG1 methylation levels were 1; therefore, this CpG was not analyzed by logistic regression. Gene-gene and gene-environment interactions were further analyzed by multifactor dimensionality reduction software 3.0.2 (MDR 3.0.2). The MDR method tests potential gene-gene or gene-environmental factor combinations to create a model that sorts cases and controls with the lowest possible classification error. The procedure is repeated 10 times to get cross-validation consistency and the number of times that a particular model is chosen as the best one and to test for balanced accuracy and the proportion of occurrences correctly classified using the model (*P* < 0.05 was statistically significant) (19). The environmental factors in the MDR model included age, body mass index (BMI), current smoking, current drinking, triglyceride (TG), high density lipoprotein (HDL), low density lipoprotein (LDL), total cholesterol, fasting blood glucose (FBG), postprandial blood glucose and HbA1c.

A path analysis of mediating effects was used to evaluate associations among baseline characteristics, gene promoter methylation level and T2DM. Because T2DM was a binary variable, we used Bayesian analysis instead of the maximum likelihood method, and the standardized regression coefficients a, b, c' and c to identify relationships between variables. (In this model the variables are a, association between independent and mediator variable in the mediating model; b, association between mediator and dependent variable in the mediating model; c', association between independent and dependent variable in the mediating model; c, association between independent and dependent variables; ab, indirect effect; c', direct effect; c, total effect; and (ab/c)^*^100%, mediating effect generated by the mediator variable in the dependent variable outcome caused by the independent variable.) In this mode, the model evaluation criterion was posterior predictive *p*-value ranging from 0 to 1 with the acceptable quantity of 0.5 or close to it (20). All analyses were conducted by SPSS 24.0 and Analysis of Moment Structures software 21.0 (AMOS 21.0) (IBM Corp, Armonk, NY, USA) (21–23).

## Results

### Population characteristics

The baseline characteristics of all participants are shown in Tables 1, 2. Compared with healthy controls, T2DM patients had a higher body mass index (BMI) (T2DM: 24.68 ± 3.46; controls: 22.20 ± 2.06, *P* < 0.001), triglyceride (TG) (T2DM: 2.15 ± 1.42; controls: 1.39 ± 0.68, *P* < 0.001) and fasting blood glucose (FBG) (T2DM: 10.21 ± 6.74; controls: 5.15 ± 0.48, *P* < 0.001) but lower high-density lipoprotein (HDL) (T2DM: 1.13 ± 0.24; controls: 1.47 ± 0.30, *P* < 0.001). T2DM with macrovascular complications, microvascular complications, and micro-macrovascular complications were more prevalent in the older patients (T2DM alone: 53.43 ± 10.39; T2DM with micro: 60.47 ± 10.78, *P* = 0.046; T2DM with macro: 63.60 ± 10.29, *P* = 0.003; T2DM with micro-macro: 64.32 ± 11.98, *P* = 0.001). Compared with T2DM patients without any complications, T2DM with micro-macrovascular complications had the longest duration of diabetes (T2DM alone: 4.64 ± 4.65; T2DM with micro-macro, 8.66 ± 5.50, *P* = 0.039), the lowest estimated glomerular filtration rate (eGFR) (T2DM alone: 93.79 ± 16.25; T2DM with micro-macro: 79.16 ± 18.97, *P* = 0.010) and the highest rate of abnormal microalbuminuria (T2DM alone: 28.57%; T2DM with micro-macro, 60.00%, *P* = 0.021).

**Table 1:** Baseline characteristics of healthy controls and T2DM patients.

**Characteristics**	**Healthy controls (*N* = 93)**	**T2DM patients (*N* = 93)**	***P*-value**
Age (years)	59.84 ± 11.65	60.23 ± 11.67	0.821^#^
Gender (male/female)	40 / 53	39 / 54	0.882^Δ^
BMI (kg/m^2^)	22.20 ± 2.06	**24.68 ± 3.46**	<0.001^**^^#^
TG (mmol/L)	1.39 ± 0.68	**2.15 ± 1.42**	<0.001^**^^∧^
HDL cholesterol (mmol/L)	1.47 ± 0.30	**1.13 ± 0.24**	<0.001^**^^#^
LDL cholesterol (mmol/L)	3.10 ± 0.66	2.96 ± 0.83	0.340^∧^
Total cholesterol (mmol/L)	5.11 ± 0.78	5.88 ± 7.59	0.663^∧^
FBG (mmol/L)	5.15 ± 0.48	**10.21 ± 6.74**	<0.001^**^^∧^
Glutamic-pyruvic transaminase (IU/L)	19.75 ± 8.28	21.98 ± 10.90	0.343^∧^
Serum creatinine (umol/L)	72.81 ± 15.52	71.24 ± 26.35	0.064^∧^
Blood uric acid (umol/L)	338.49 ± 84.61	365.37 ± 113.80	0.186^∧^
Postprandial blood glucose (mmol/L)	NA	15.61 ± 4.59	NA
HbA1c (%)	NA	9.37 ± 2.71	NA
Fasting C-peptide (ng/ml)	NA	1.53 ± 1.26	NA
Postprandial 1 h C-peptide (ng/ml)	NA	2.61 ± 1.90	NA
Postprandial 2 h C-peptide (ng/ml)	NA	3.95 ± 3.10	NA
eGFR (mL/min)	NA	80.93 ± 19.75	NA
Abnormal-microalbuminuria [n(%)]	NA	37 (20.43%)	NA
Duration of diabetes (years)	0	6.76 ± 5.86	NA
Current smoking [n(%)]	NA	23 (24.73%)	NA
Current drinking [n(%)]	NA	6 (6.45%)	NA
Only Oral DM medication [n(%)]	NA	19 (20.43%)	NA
Only Insulin treatment [n(%)]	NA	13 (13.98%)	NA
Both Oral DM medication and insulin treatment [n(%)]	NA	60 (64.52%)	NA

*Data are shown as the means ± standard deviation or number (%)*.

** *P < 0.001; the bold in the table means statistically significant. (P < 0.05)*.

# *The P-value of T-test*,

Δ *the P-value of χ2 test; the P-values of Mann-Whitney test*.

*T2DM, type 2 diabetes mellitus; BMI, body mass index; TG, triglyceride; HDL, high density lipoprotein; LDL, low density lipoprotein; FBG, fasting blood glucose; HbA1c, glycated hemoglobin; eGFR, estimated glomerular filtration rate; NA, not available*.

**Table 2:** Baseline characteristics of T2DM subgroups patients.

**Characteristics**	**T2DM without any complication (*n* = 28)**	**T2DM with microvascular complications^**a**^ (*n* = 15)**	***P*-value**	**T2DM with macrovascular complications^**a**^ (*n* = 25)**	***P*-value**	**T2DM with both microvascular and macrovascular complications^**a**^ (*n* = 25)**	***P*-value**
Age (years)	53.43 ± 10.39	**60.47 ± 10.78**	0.046^*^^#^	**63.60 ± 10.29**	0.003^*^^#^	**64.32 ± 11.98**	0.001^*^^#^
Gender (male/female)	16/12	5/10	0.137^Δ^	9/16	0.124^Δ^	9/16	0.124^Δ^
BMI (kg/m^2^)	23.77 ± 3.42	23.61 ± 3.63	0.998**^#^**	25.04 ± 3.07	0.444**^#^**	25.97 ± 3.51	0.077**^#^**
TG (mmol/L)	2.50 ± 1.90	1.55 ± 0.66	0.186**^∧^**	2.06 ± 1.24	1.000**^∧^**	2.21 ± 1.23	1.000**^∧^**
HDL cholesterol (mmol/L)	1.12 ± 0.18	1.06 ± 0.31	0.851**^#^**	1.16 ± 0.27	0.875**^#^**	1.15 ± 0.23	0.909**^#^**
LDL cholesterol (mmol/L)	2.82 ± 0.75	2.85 ± 0.60	1.000**^∧^**	2.92 ± 0.92	1.000**^∧^**	3.23 ± 0.94	0.276**^∧^**
Total cholesterol (mmol/L)	5.10 ± 1.09	4.60 ± 0.88	0.516**^∧^**	8.02 ± 14.68	1.000**^∧^**	5.45 ± 1.19	0.747**^∧^**
FBG (mmol/L)	8.97 ± 3.91	9.07 ± 2.78	1.000**^∧^**	11.49 ± 10.12	1.000**^∧^**	11.13 ± 6.15	0.414**^∧^**
Glutamic-pyruvic transaminase (IU/L)	27.21 ± 12.28	19.00 ± 10.40	0.930**^∧^**	20.86 ± 11.10	0.138**^∧^**	19.63 ± 8.00	0.090**^∧^**
Serum creatinine (umol/L)	67.21 ± 24.81	71.00 ± 22.74	1.000**^∧^**	70.00 ± 32.76	1.000**^∧^**	77.33 ± 23.38	0.204**^∧^**
Blood uric acid (umol/L)	347.00 ± 103.20	326.20 ± 107.65	0.711**^∧^**	360.70 ± 98.74	1.000**^∧^**	415.00 ± 130.93	0.072**^∧^**
Postprandial blood glucose (mmol/L)	15.98 ± 4.92	16.51 ± 4.11	0.978**^#^**	15.34 ± 4.66	0.941**^#^**	14.80 ± 4.61	0.768**^#^**
HbA1c (%)	9.53 ± 2.86	9.80 ± 2.21	0.984**^#^**	9.61 ± 3.23	0.999**^#^**	8.56 ± 2.05	0.550**^#^**
Fasting C-peptide (ng/ml)	1.48 ± 1.35	0.83 ± 0.38	0.339**^#^**	1.57 ± 0.90	0.990**^#^**	1.95 ± 1.65	0.464**^#^**
Postprandial 1 h C-peptide (ng/ml)	3.04 ± 2.26	1.79 ± 0.78	0.360**^#^**	2.87 ± 1.87	0.991**^#^**	1.68 ± 1.54	0.397**^#^**
Postprandial 2 h C-peptide (ng/ml)	3.45 ± 2.75	2.44 ± 1.29	0.658**^#^**	5.47 ± 3.72	0.077**^#^**	3.76 ± 2.99	0.979**^#^**
eGFR (mL/min)	93.79 ± 16.25	78.26 ± 15.05	0.117**^#^**	79.16 ± 15.05	0.067**^#^**	**79.16 ± 18.97**	0.010^*^^#^
Abnormal-microalbuminuria [n(%)]	8 (28.57%)	6 (40.00%)	0.446^Δ^	8 (32.00%)	0.768^Δ^	**15 (60.00%)**	0.021^*^^Δ^
Duration of diabetes (years)	4.64 ± 4.65	6.22 ± 4.81	0.753**^#^**	7.59 ± 7.39	0.184^Δ^	**8.66 ± 5.50**	0.039^*^^#^
Current smoking [n(%)]	9 (32.14%)	4 (26.67%)	0.709^Δ^	4 (16.00%)	0.173^Δ^	6 (24.00%)	0.511^Δ^
Current drinking [n(%)]	3 (10.71%)	1 (6.67%)	0.663^Δ^	1 (4.00%)	0.356^Δ^	1 (4.00%)	0.356^Δ^
Only Oral DM medication [n(%)]	4 (14.29%)	1 (6.67%)	0.458^Δ^	6 (24.00%)	0.367^Δ^	8 (32.00%)	0.124^Δ^
Only Insulin treatment [n(%)]	6 (21.43%)	3 (20.00%)	0.913^Δ^	1 (4.00%)	0.061^Δ^	3 (12.00%)	0.361^Δ^
Both Oral DM medication and insulin treatment [n(%)]	17 (60.71%)	11 (73.33%)	0.408^Δ^	18 (72.00%)	0.386^Δ^	14 (56.00%)	0.728^Δ^

*Data are shown as the means ± standard deviation or number (%)*.

a *vs. T2DM patients without any complication*;

* *P < 0.05; The bold in the table means statistically significant. (P < 0.05)*.

# *The P-value of One-way analysis of variance (ANOVA) and the Dunnett method*,

Δ *the P-value of χ^2^ test and Bonferroni correction*;

∧ *the P of Kruskal-Wallis test and Bonferroni's correction*.

*T2DM, type 2 diabetes mellitus; BMI, body mass index; TG, triglyceride; HDL, high density lipoprotein; LDL, low density lipoprotein; FBG, fasting blood glucose; HbA1c, glycated hemoglobin; eGFR, estimated glomerular filtration rate*.

### Lower methylation levels in AIM2 promoter increased the risk of T2DM

**Figure 1:**
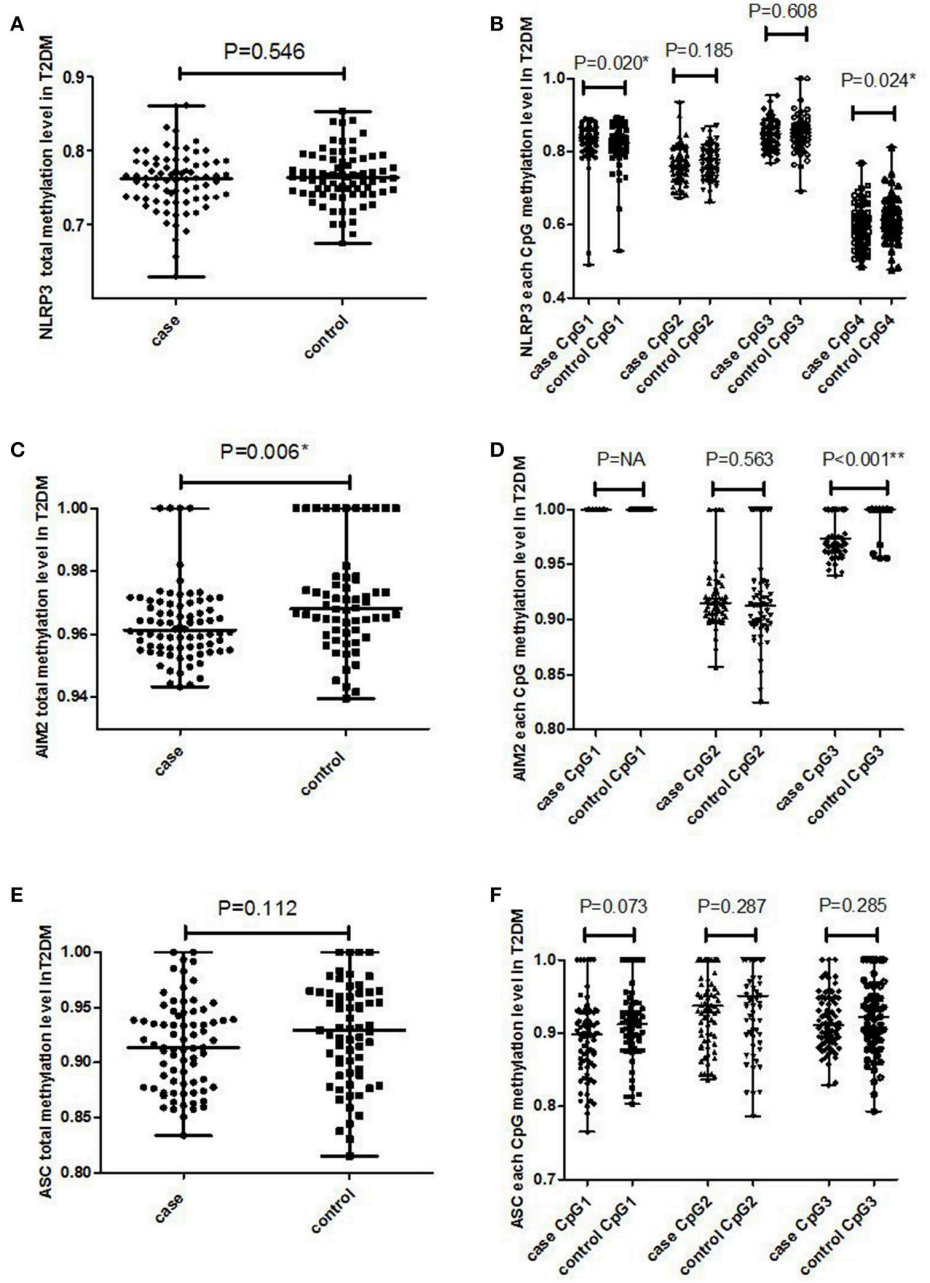
The methylation level of promotor region in NLRP3, AIM2, and ASC genes in T2DM cases and healthy controls. **(A)** Total methylation level in NLRP3 gene; **(B)** each CpG methylation level in NLRP3 gene; **(C)** total methylation level in AIM2 gene; **(D)** each CpG methylation level in AIM2 gene; **(E)** total methylation level in ASC gene; **(F)** each CpG methylation level in ASC gene. The Mann-Whitney test ^*^P < 0.05, ^**^P < 0.001.

No significant difference was found in the total methylation level of the NLRP3 promoter region between cases and controls (*P* = 0.546), whereas a significant difference was observed in NLRP3 CpG1 and CpG4 (*P* = 0.020, *P* = 0.024) (Figures 1A,B). The total methylation and AIM2 CpG3 methylation in the AIM2 promoter were statistically lower in cases than controls (Figures 1C,D) (*P* = 0.006). Neither total methylation nor any CpG methylation in the ASC promoter region was found to be associated with T2DM (Figures 1E,F) (total *P* = 0.112, CpG1 *P* = 0.073, CpG2 *P* = 0.287, CpG3 *P* = 0.285). In the logistic regression model with adjusted BMI (Table 3), no significant difference was observed in NLRP3 and ASC methylation level (both total methylation and each CpG methylation) between T2DM cases and controls (*P* > 0.05). However, healthy individuals with lower AIM2 total methylation and AIM2 CpG3 methylation level (<methylation median) exhibited 2.29-fold (*P* = 0.011) and 8.29-fold (*P* < 0.001) increased risks of developing T2DM compared to those with higher methylation levels (≧ methylation median), respectively.

**Table 3:** Association of NLRP3, AIM2, and ASC genes promotor methylation with T2DM.

**Gene**	**CpGs**	**Methylation level**^a^****	**T2DM patients vs. healthy controls**	
			**OR (95% CI)^**b**^**	***P*-value^**b**^**
NLRP3	Total methylation	≧0.764	Ref.	
		<0.764	1.08 (0.52~2.24)	0.804
		Per SD less methylation	1.14 (0.78~1.65)	0.499
	CpG1	≧0.823	Ref.	
		<0.823	0.47 (0.22~1.02)	0.056
		Per SD less methylation	0.98 (0.68~1.43)	0.924
	CpG2	≧0.778	Ref.	
		<0.778	1.77 (0.84~3.73)	0.132
		Per SD less methylation	1.14 (0.78~1.66)	0.498
	CpG3	≧0.851	Ref.	
		<0.851	0.99 (0.48~2.07)	0.988
		Per SD less methylation	0.97 (0.66~1.41)	0.860
	CpG4	≧0.607	Ref.	
		<0.607	1.44 (0.69~3.03)	0.332
		Per SD less methylation	1.37 (0.92~2.04)	0.118
AIM2	Total methylation	≧0.968	Ref.	
		<0.968	**2.29 (1.28~6.66)**	0.011^*^
		Per SD less methylation	1.40 (0.94~2.07)	0.096
	CpG2	≧0.911	Ref.	
		<0.911	0.91 (0.42~1.97)	0.808
		Per SD less methylation	1.16 (0.80~1.68)	0.446
	CpG3	=1.000	Ref.	
		<1.000	**8.29 (3.37~20.40)**	<0.001^**^
		Per SD less methylation	**1.72 (1.06~2.80)**	0.029^*^
ASC	Total methylation	≧0.929	Ref.	
		<0.929	1.21 (0.56~2.61)	0.621
		Per SD less methylation	1.10 (0.75~1.59)	0.641
	CpG1	≧0.912	Ref.	
		<0.912	1.35 (0.62~2.93)	0.453
		Per SD less methylation	1.17 (0.80~1.69)	0.426
	CpG2	≧0.951	Ref.	
		<0.951	1.14 (0.53~2.43)	0.741
		Per SD less methylation	0.96 (0.66~1.41)	0.844
	CpG3	≧0.922	Ref.	
		<0.922	1.30 (0.61~2.78)	0.504
		Per SD less methylation	1.13 (0.78~1.64)	0.511

a *Median of total or each CpG methylation in healthy controls*.

b *OR, P-values were from logistic regression analyses controlling for BMI*.

* *P < 0.05*,

** *P < 0.001; The bold in the table means statistically significant. (P < 0.05)*.

*Per SD less methylation, the decrease of per standard deviation of CpG methylation, the change of OR value*.

### Lower methylation levels in NLRP3, AIM2 and ASC promoters increased the risk of T2DM vascular complications

**Figure 2:**
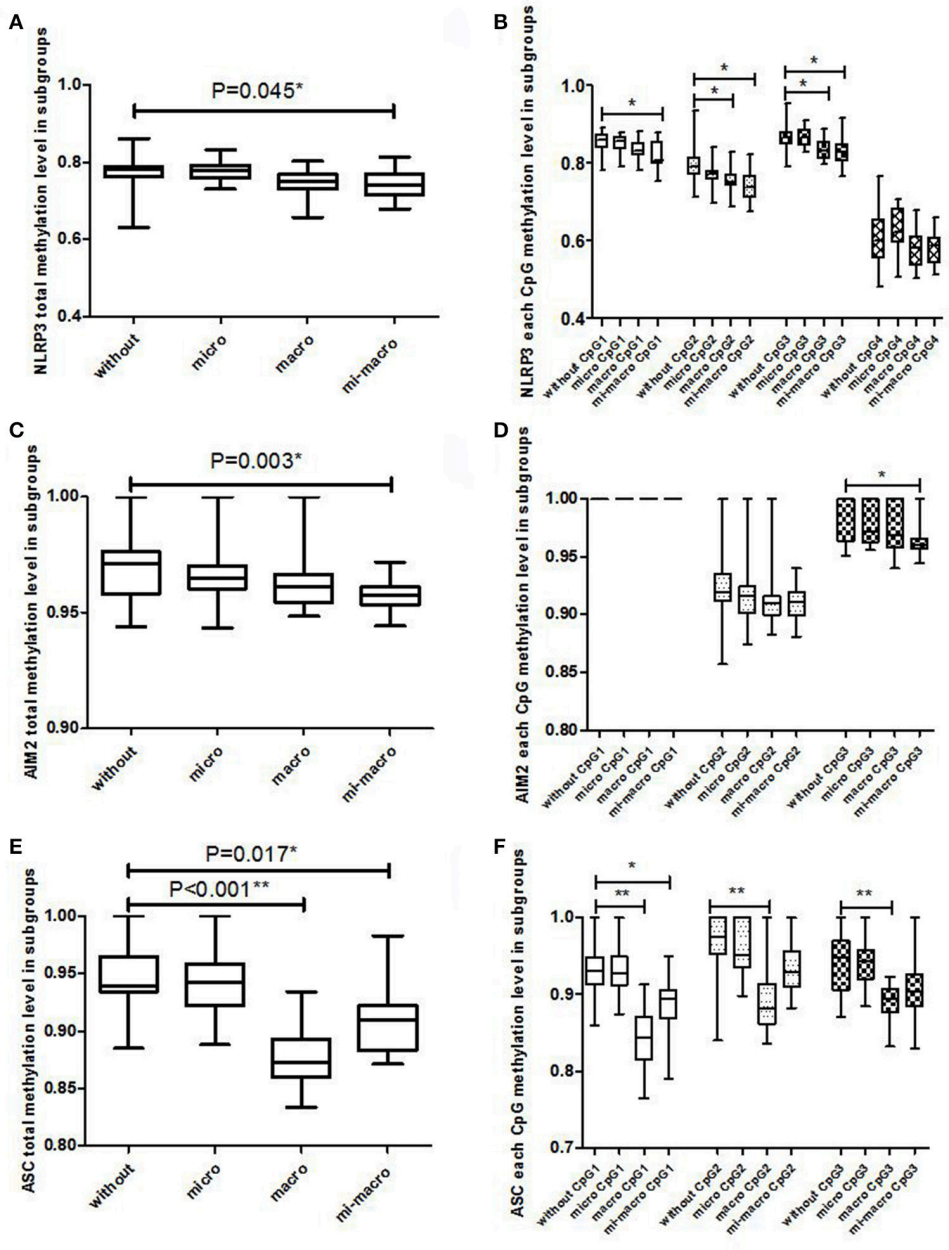
The association between promoter methylation in NLRP3, AIM2, and ASC genes and T2DM complication (T2DM without any complication vs. T2DM with microvascular complications; T2DM without any complication vs. T2DM with macrovascular complications; T2DM without any complication vs. T2DM with both microvascular and macrovascular complications). **(A)** NLRP3 total methylation level; **(B)** NLRP3 each CpG methylation level; **(C)** AIM2 total methylation level; **(D)** AIM2 each CpG methylation level; **(E)** ASC total methylation level; **(F)** ASC each CpG methylation level. The Kruskal-Wallis test was used among T2DM subgroups and a Dunn-Bonferroni test for *post-hoc* comparisons, * Bonferroni-corrected *P* < 0.05, ** Bonferroni-corrected *P* < 0.001.

As shown in Figure 2, the total methylation level in the NLRP3, AIM2 and ASC promoter regions, NLRP3 CpG1, NLRP3 CpG2, NLRP3 CpG3, AIM2 CpG3, and ASC CpG1 methylation in T2DM without any complications were significantly higher than T2DM with micro-macrovascular complications (*P* < 0.05). We also found that the total methylation in ASC, NLRP3 CpG2, NLRP3 CpG3 and three ASC CpGs methylations in T2DM patients without any complication were higher than in T2DM patients with macrovascular complications (*P* < 0.05).

In the logistic regression model, after controlling for age (Table 4), T2DM patients without any vascular complications when carrying lower methylation levels in NLRP3 CpG2 and AIM2 had 6.45 and 9.48 times higher risk (*P* = 0.045, *P* = 0.038) of developing diabetic microvascular complications than T2DM patients with higher methylation, respectively.

**Table 4:** Association of NLRP3, AIM2, and ASC genes promotor methylation with T2DM vascular complications.

**Gene**	**CpGs**	**Methylation level^**a**^**	**T2DM with microvascular complications vs. T2DM without any complication**		**T2DM with macrovascular complications vs. T2DM without any complication**		**T2DM with both micro-macrovascular complications vs. T2DM without any complication**	
			**OR (95% CI)^**b**^**	***P*-value^**b**^**	**OR (95% CI)^**b**^**	***P*-value^**b**^**	**OR (95% CI)^**b**^**	***P*-value^**b**^**
NLRP3	Total methylation	≧0.782	Ref.		Ref.		Ref.	
		< 0.782	1.11 (0.26~4.78)	0.890	**36.03 (3.11~417.06)**	0.004^*^	**17.78 (2.04~155.28)**	0.009^*^
		Per SD less methylation	1.21 (0.56~2.63)	0.623	2.00 (0.88~4.55)	0.098	**2.19 (1.01~4.76)**	0.048^*^
	CpG1	≧0.859	Ref.		Ref.		Ref.	
		< 0.859	1.06 (0.25~4.57)	0.936	3.83 (0.82~17.87)	0.088	4.34 (0.83~22.73)	0.083
		Per SD less methylation	1.20 (0.50~2.87)	0.684	1.17 (0.69~1.99)	0.568	1.39 (0.71~2.72)	0.338
	CpG2	≧0.790	Ref.		Ref.		Ref.	
		< 0.790	**6.45 (1.05~39.78)**	0.045^*^	**14.90 (1.98~111.96)**	0.009^*^	**17.78 (2.06~155.28)**	0.009^*^
		Per SD less methylation	0.60 (0.24~1.49)	0.266	**3.98 (1.23~12.95)**	0.022^*^	**3.75 (1.38~10.18)**	0.010^*^
	CpG3	≧0.865	Ref.		Ref.		Ref.	
		< 0.865	0.69 (0.15~3.26)	0.641	**6.09 (1.13~32.78)**	0.036^*^	3.46 (0.71~16.93)	0.125
		Per SD less methylation	1.18 (0.51~2.77)	0.696	**4.07 (1.37~12.05)**	0.011^*^	**3.22 (1.27~8.16)**	0.014^*^
	CpG4	≧0.600	Ref.		Ref.		Ref.	
		< 0.600	0.27 (0.05~1.43)	0.123	2.69 (0.64~11.38)	0.178	2.73 (0.59~12.74)	0.202
		Per SD less methylation	1.65 (0.82~3.30)	0.160	1.47 (0.69~3.14)	0.317	1.65 (0.72~3.80)	0.238
AIM2	Total methylation	≧0.971	Ref.		Ref.		Ref.	
		< 0.971	**9.48 (1.14~79.00)**	0.038^*^	**30.90 (2.59~368.49)**	0.007^*^	**306.22 (2.29~1729.76)**	0.006^*^
		Per SD less methylation	0.58 (0.27~1.24)	0.157	**3.16 (1.25~7.97)**	0.015^*^	**9.55 (1.97~46.24)**	0.005^*^
	CpG2	≧0.919	Ref.		Ref.		Ref.	
		< 0.919	2.51 (0.45~14.07)	0.294	**7.46 (1.28~43.37)**	0.025^*^	**5.91 (1.07~32.71)**	0.042^*^
		Per SD less methylation	0.65 (0.32~1.29)	0.215	**2.91 (1.13~7.55)**	0.028^*^	**3.40 (1.05~10.99)**	0.041^*^
	CpG3	≧1.000	Ref.		Ref.		Ref.	
		< 1.000	2.66 (0.05~12.48)	0.226	4.41 (0.94~20.61)	0.059	**257.63 (5.34~1429.38)**	0.005^*^
		Per SD less methylation	0.67 (0.30~1.48)	0.321	1.94 (0.93~4.08)	0.080	**8.27 (2.24~30.55)**	0.002^*^
ASC	Total methylation	≧0.939	Ref.		Ref.		Ref.	
		< 0.939	1.00 (0.23~4.26)	0.996	NA	NA	2.60 (0.49~13.83)	0.262
		Per SD less methylation	0.80 (0.28~2.30)	0.686	**35.78 (3.67~349.19)**	0.002^*^	**4.43 (1.19~16.45)**	0.026^*^
	CpG1	≧0.931	Ref.		Ref.		Ref.	
		< 0.931	2.62 (0.55~12.40)	0.225	NA	NA	**12.69 (1.24~130.05)**	0.032^*^
		Per SD less methylation	1.04 (0.37~2.88)	0.943	**69.22 (4.05~1620.25)**	0.003^*^	**10.86 (1.57~75.11)**	0.016^*^
	CpG2	≧0.975	Ref.		Ref.		Ref.	
		< 0.975	2.74 (0.57~13.14)	0.209	**38.91(2.26~1183.45)**	0.012^*^	2.33 (0.47~11.64)	0.302
		Per SD less methylation	0.53 (0.16~1.72)	0.290	**8.62 (2.31~32.15)**	0.011^*^	2.45 (0.84~7.13)	0.101
	CpG3	≧0.949	Ref.		Ref.		Ref.	
		< 0.949	1.72 (0.39~7.62)	0.474	NA	NA	**11.28 (1.13~112.33)**	0.039^*^
		Per SD less methylation	1.01 (0.46~2.24)	0.980	5.74 (1.69~19.47)	0.005^*^	1.92 (0.85~4.35)	0.119

a *Median of total or each CpG methylation in T2DM without any complications*.

b *OR, P-values were from logistic regression analyses controlling for age*.

* *P < 0.05; The bold in the table means statistically significant. (P < 0.05)*.

*NA, not available because of zero cases in case group, grouped by methylation median of control group; Per SD less methylation, the decrease of per standard deviation of CpG methylation, the change of OR value*.

A lower total methylation level in the NLRP3 and AIM2 promoters and the risk of diabetic macrovascular complications was also observed, with 36.03 and 30.90 times increases in susceptibility (*P* = 0.004, *P* = 0.007), respectively. Moreover, T2DM patients with lower methylation levels in NLRP3 CpG2, NLRP3 CpG3, AIM2 CpG2, and ASC methylation levels were more susceptible to developing macrovascular complications (*P* < 0.05). Similar associations of lower methylation level in NLRP3, AIM2 and ASC promoters and diabetic micro-macrovascular complications were observed (*P* < 0.05).

However, in the MDR analysis that explored the best gene-gene interaction models and the best gene-environment interaction models among NlRP3, AIM2, and ASC promoter methylation and environmental factors, no interaction effect was identified for the susceptibility of T2DM and its vascular complications (Table S3) (*P* > 0.05).

### The methylation level in ASC CPG1 or CPG3 had partial mediating effects between age and T2DM vascular complications

**Figure 3:**
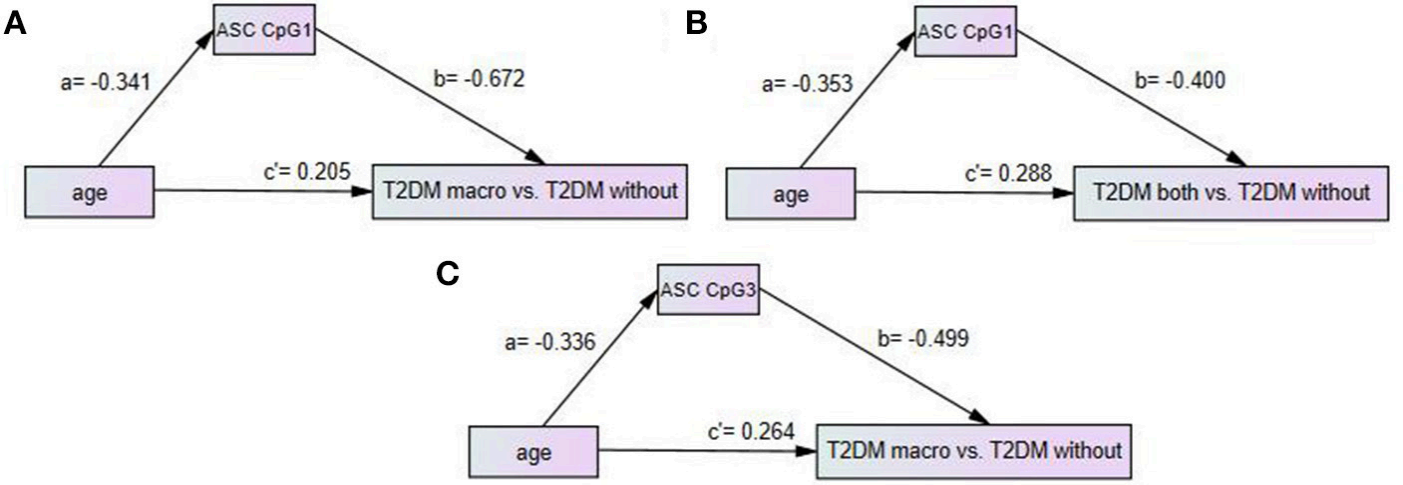
Path model analysis with standardized coefficients of age, ASC CpGs methylation level and T2DM with vascular complications. **(A)** Path model of age, ASC CpG1 and T2DM with macrovascular complications vs. T2DM without any complication; **(B)** Path model of age, ASC CpG1 and T2DM with both microvascular and macrovascular complications vs. T2DM without any complication; **(C)** Path model of age, ASC CpG3 and T2DM macrovascular complications vs. T2DM without any complication. AOMS Bayesian analysis method was used in path model analysis.

There was a significant difference in age between T2DM without any vascular complications and T2DM vascular complications. Thus, we used the path analysis method to explore the mediating effect among the variables of age, gene promoter methylation level and T2DM vascular complications. The posterior predictive *p*-values in all three models of Figure 3 were all 0.47, closed to 0.5, so the models were effective. The standardized coefficient in the model was considered to have a significant effect when the 95% CI did not include 0. The partial mediating model was effective with all three effective standardized coefficients, which meant that the ASC CpG methylation level had a partial mediating effect between age and complications (Figure 3).

A negative correlation existed for age and ASC CpG1 methylation level (a = −0.341, 95% CI: −0.575 ~ −0.068), and a negative correlation existed for ASC CpG1 methylation level and T2DM macrovascular complications (b = −0.672, 95% CI: −0.816~-0.484). A positive correlation existed for age and diabetic macrovascular complications (c' = 0.205, 95% CI: 0.007~0.401). The indirect effect (ab) of age on macrovascular complications via ASC CPG1 was 0.229. The direct effect (c') of age on macrovascular complications was 0.205. The total effect (c) of age on macrovascular complications was 0.434 (c = 0.434, 95% CI: 0.190~0.636). The mediating effect (ab/c)^*^100% of a low methylation level in ASC CPG1 was 52.8%, which could explain 52.8% of the total effect of age on the macrovascular complications of type 2 diabetes. That is to say, the older the T2DM patients are, the greater the risk of macrovascular complications, and age could increase the risk of complications under the lower methylation level in ASC CPG1.

A negative correlation existed for age and ASC CpG1 methylation level (a = −0.353, 95% CI: −0.577 ~ −0.062), and a negative correlation existed for ASC CpG1 methylation level and T2DM macro-microvascular complications (b = −0.400, 95% CI: −0.644 ~ −0.099). A positive correlation existed for age and diabetic macro-microvascular complications (c' = 0.288, 95% CI: 0.036~0.523). The indirect effect (ab) of age on macro-microvascular complications via ASC CPG1 was 0.141. The direct effect (c') of age on macro-microvascular complications was 0.288. The total effect (c) of age on macro-microvascular complications was 0.429 (c = 0.429, 95% CI: 0.181~0.633). The low methylation level in ASC CPG1 had a mediating effect (ab/c)^*^100% during macro-microvascular complications caused by higher age of 32.9%, which meant that the older the patients were, the higher the risk of the complications was, and age increased the risk of complications by lower methylation in ASC CPG1 at the same time.

A negative correlation existed for age and ASC CpG3 methylation level (a = −0.336, 95% CI: −0.575 ~ −0.057), and a negative correlation existed for ASC CpG3 methylation level and T2DM macrovascular complications (b = −0.499, 95% CI: −0.700 ~ −0.248), while a positive correlation existed for age and diabetic macrovascular complications (c' = 0.264, 95% CI: 0.036~0.485). The indirect effect (ab) of age on macrovascular complications via ASC CPG3 was 0.168. The direct effect (c') of age on macrovascular complications was 0.264. The total effect (c) of age on macrovascular complications was 0.432 (c = 0.432, 95% CI: 0.188~0.636). A low ASC CPG3 methylation level had a mediating effect (ab/c)^*^100% during macrovascular complications caused by higher age of 38.9%. Older age and lower methylation level in ASC CPG3 could work together to give rise to the risk of macrovascular complications.

## Discussion

Several studies had demonstrated that some inflammation-related genes such as NLRP3, AIM2, and ASC were associated with T2DM and its vascular complications; however, epigenetics, especially DNA methylation, were not studied (3, 4, 24). In our study, we found that the total methylation level of the AIM2 promoter in T2DM patients in a Southern Han Chinese population was lower than that in healthy controls. T2DM patients carrying a lower methylation level in NLRP3, AIM2, and ASC gene promoters could have a higher risk of developing vascular complications. The age of T2DM patients with vascular complications was older than that of T2DM patients without any complications. ASC CpG1 or CpG3 partially mediated the effect between age and T2DM vascular complications.

In the clinical baseline characteristics analysis, BMI and TG in T2DM patients were significantly higher than those in healthy controls, which was consistent with the study of Shai et al. (25). In addition, a higher BMI was associated with increased blood glucose in T2DM (26). Jensen M D et al. found that obesity factors, not only BMI but also the quality and distribution of fat mass, played important roles in chronic metabolic diseases, such as T2DM (27). In our future studies, we should collect waist-hip ratios to get an association that is more accurate. In our study, the HDL of T2DM patients was lower than that in healthy controls. The evidence from Xu et al. (28) and Basta et al. (29) showed that HDL could inhibit β cell apoptosis, increasing β cell function and glucose uptake of skeletal muscle. Therefore, increasing the level of HDL could reduce the risk of T2DM.

NLRP3, AIM2, and ASC have been found to be related to T2DM, playing a key role in the process of T2DM induced by abnormal glucose metabolism, and would further result in a series of diabetic complications such as vascular complications (24, 30, 31). Higher expression of NLRP3, AIM2, and ASC could give rise to IL-1β and IL-18 secretion, promoting atherosclerosis and further destabilizing atherosclerotic plaques, which indicates their importance in cardiovascular disease pathogenesis (32, 33). Inflammation from continuously increasing IL-1β and IL-18 in diabetes patients could also lead to retinal and kidney lesions (34, 35). NLRP3, IL-1β, and IL-18 in rats with diabetic nephropathy were markedly increased (36).

DNA methylation was linked to T2DM pathogenesis by regulating insulin gene expression and damaging insulin secretion (37). The change in methylation level in the gene promoter also alters gene transcription (11, 38). In our logistic regression with covariants, the reduction in total methylation or some CpGs methylation in the NLRP3, AIM2 and ASC promoters increased the risk of diabetes or diabetic vascular complications to a varying degree. Although no research related to the association between AIM2 methylation and diabetic vascular complications was found, AIM2 methylation level changes could affect the relationship between C-reactive protein and traumatic stress disorder (39). We should conduct a future study on a large sample concerning the relationship between AIM2 methylation and diabetic vascular complications. In addition, similar studies of multiethnic populations all over the world are needed. Haldar et al. (40) reported that there was high NLRP3 methylation and low expression in human chronic bladder inflammatory tissue. Human embryonic kidney cells infected with *Mycobacterium tuberculosis* H37Rv reduced NLRP3 promoter region methylation and then activated NLRP3 inflammation, resulting in upregulation of the RNA level of NLRP3 and IL-1β (41). The VO2 max of heart failure patients was positively related to the level of the ASC average methylation level and negatively correlated with the plasma concentration of IL-1β (42). These studies indirectly supported our observations of relationships between NLRP3, AIM2, and ASC methylation level changes and inflammation. We should enlarge the study sample size and carry out extensive experimental research in a future study to verify the association between NLRP3, AIM2, and ASC promoter methylation and T2DM and its vascular complications, which could provide a new perspective for genetic marker screening of T2DM.

In the MDR analysis, we did not identify any gene-gene or gene-environment interactions in T2DM and its vascular complications. We should collect more demographic data in the next study (such as smoking and drinking of healthy controls). In the path analysis, the mediating models showed that ASC CpG1 or ASC CpG3 had partial mediating effects on age and T2DM vascular complications, which indicated that not only could higher age directly promote the risk of diabetic vascular complications, but it also could indirectly increase the risk of complications via a lower ASC CpG methylation level. Chung et al. (43) demonstrated that an older population had a lower level of ASC methylation, and ASC was an inflammatory gene closely related to age in some ways. Peripheral blood ASC methylation decreased with age, while a certain level of exercise intervention attenuated this age-related decrease (7). DNA methylation (such as ARHGEF25, CD2485, and IVRT) was also associated with age and cardiovascular disease (44). Thus, age and ASC CpG methylation might be the vital risk factor for T2DM vascular complications; however, further mechanistic investigation should be performed.

In conclusion, there was a close association between NLRP3, AIM2 and ASC promoter methylation and T2DM and vascular complications. This study provides new epidemiological clues about the crucial role of T2DM gene marker screening and clinical prevention.

## Ethics statement

This study was carried out in accordance with the Ethics Committee of Jinan University with written informed consent from all subjects. All subjects gave written informed consent in accordance with the Declaration of Helsinki. The protocol was approved by the Ethics Committee of Jinan University.

## Author contributions

ZZ, LW, and ZW contributed equally to the writing of this paper. LN provided all sample of this study. SH, SZ, XY, DL, JW, XZ, YL, YW, SD, XH, XD, KZ, XC, SH, CZ, YH, and BZ carried out data collection, the extraction of DNA and the bisulfite conversion. GY and CJ carried out whole design.

### Conflict of interest statement

The authors declare that the research was conducted in the absence of any commercial or financial relationships that could be construed as a potential conflict of interest.
