# Supplementary material for: Association Analysis of NLRP3 Inflammation-Related Gene Promotor Methylation as Well as Mediating Effects on T2DM and Vascular Complications in a Southern Han Chinese Population
Source: Front Endocrinol (Lausanne). 2018 Nov 29;9:709. doi: 10.3389/fendo.2018.00709 (PMC6281743; doi:10.3389/fendo.2018.00709)
Supplement: Supplementary file 1 [file Table_1.DOCX]

**Supplementary materials**

Table S1. Primer sequences of NLRP3, AIM2 and ASC genes for promotor methylation

| Primer name | primer sequences (5’-3’) | Product size (bp) | CpG number |
| --- | --- | --- | --- |
| NLRP3 | F: ATATTTTTTTATTGAATTGGGTGTT  R: AAAAAAACACACACAAACCTACTCTAC | 157 | 4 |
| AIM2 | F: ATGAGTTGGTTAATTTAAGGTAAAATGTT  R: AATTACATAATAAAACAAACTCCTC | 290 | 3 |
| ASC | F: GGTTTTAAATTTTTGATTATTTATTT  R: TTATACCACTACACTCTATACCACTC | 198 | 3 |

Table S2. Methylation median of NLRP3, AIM2 and ASC genes promotor in healthy controls and T2DM without any complication

| Gene | CpGs | Methylation median of healthy controls | Methylation median of T2DM without any complication |
| --- | --- | --- | --- |
| NLRP3 | Total methylation | 0.764 | 0.782 |
|  | CpG1 | 0.823 | 0.859 |
|  | CpG2 | 0.778 | 0.790 |
|  | CpG3 | 0.851 | 0.865 |
|  | CpG4 | 0.607 | 0.600 |
| AIM2 | Total methylation | 0.968 | 0.971 |
|  | CpG1 | 1.000 | 1.000 |
|  | CpG2 | 0.911 | 0.919 |
|  | CpG3 | 1.000 | 1.000 |
| ASC | Total methylation | 0.929 | 0.939 |
|  | CpG1 | 0.912 | 0.931 |
|  | CpG2 | 0.951 | 0.975 |
|  | CpG3 | 0.922 | 0.949 |

Table S3. MDR interaction models for T2DM and its vascular complications

| Best candidate model | Testing balance accuracy | P value | Cross validation consistency |
| --- | --- | --- | --- |
| Healthy controls vs. T2DM |  |  |  |
| Gene-gene promotor total methylation |  |  |  |
| ASC | 0.509 | 0.483 | 10/10 |
| NLRP3*AIM2 | 0.505 | 0.948 | 8/10 |
| NLRP3*AIM2*ASC | 0.522 | 0.741 | 10/10 |
|  |  |  |  |
|  |  |  |  |
| Environment-gene promotor total methylation |  |  |  |
| FBG | 0.651 | 0.304 | 10/10 |
| NLRP3*FBG | 0.502 | 0.972 | 10/10 |
| NLRP3*AIM2*FBG | 0.495 | 0.450 | 9/10 |
|  |  |  |  |
| T2DM without any complication vs.  T2DM microvascular complications |  |  |  |
|  |  |  |  |
| Gene-gene promotor total methylation |  |  |  |
| ASC | 0.483 | 0.948 | 4/10 |
| NLRP3*ASC | 0.558 | 0.777 | 8/10 |
| NLRP3*AIM2*ASC | 0.538 | 0.814 | 10/10 |
|  |  |  |  |
| Environment-gene promotor total methylation |  |  |  |
| TG | 0.520 | 0.891 | 7/10 |
| NLRP3*BMI | 0.469 | 0.833 | 7/10 |
| NLRP3*AIM2*age | 0.502 | 0.985 | 9/10 |
|  |  |  |  |
| T2DM without any complication vs.  T2DM macrovascular complications |  |  |  |
|  |  |  |  |
| Gene-gene promotor total methylation |  |  |  |
| ASC | 0.619 | 0.948 | 10/10 |
| AIM2*ASC | 0.585 | 0.777 | 7/10 |
| NLRP3*AIM2*ASC | 0.551 | 0.814 | 10/10 |
|  |  |  |  |
| Environment-gene promotor total methylation |  |  |  |
| FBG | 0.516 | 0.876 | 10/10 |
| NLRP3*age | 0.498 | 0.833 | 10/10 |
| NLRP3*AIM2*age | 0.480 | 0.736 | 9/10 |
|  |  |  |  |
| T2DM without any complication vs.  T2DM micro-macrovascular complications |  |  |  |
|  |  |  |  |
| Gene-gene promotor total methylation |  |  |  |
| AIM2 | 0.574 | 0.722 | 6/10 |
| NLRP3*AIM2 | 0.625 | 0.396 | 10/10 |
| NLRP3*AIM2*ASC | 0.571 | 0.534 | 10/10 |
|  |  |  |  |
| Environment-gene promotor total methylation |  |  |  |
| FBG | 0.498 | 0.979 | 8/10 |
| NLRP3*AIM2 | 0.625 | 0.396 | 10/10 |
| NLRP3*AIM2*ASC | 0.571 | 0.534 | 10/10 |

Table S4. The normality test of quantitative variables in healthy control group and T2DM group

| Characteristics | Shapiro-Wilk Test for Normality of control group | | | | Shapiro-Wilk Test for Normality of  T2DM group | | | |
| --- | --- | --- | --- | --- | --- | --- | --- | --- |
|  |  | statistic | P value |  |  | statistic | P value |  |
| Age (years) | 0.975 | | 0.069 | | 0.975 | | 0.069 | |
| BMI (kg/m^2^) | 0.992 | | 0.908 | | 0.998 | | 0.661 | |
| TG (mmol/L) | 0.881 | | **<0.001**** | | 0.814 | | **<0.001**** | |
| HDL cholesterol (mmol/L) | 0.983 | | 0.270 | | 0.979 | | 0.151 | |
| LDL cholesterol (mmol/L) | 0.963 | | **0.009*** | | 0.989 | | 0.674 | |
| Total cholesterol (mmol/L) | 0.963 | | **0.009*** | | 0.176 | | **<0.001**** | |
| FBG (mmol/L) | 0.981 | | 0.210 | | 0.638 | | **<0.001**** | |
| Glutamic-pyruvic transaminase (IU/L) | 0.882 | | **<0.001**** | | 0.916 | | **<0.001**** | |
| Serum creatinine (umol/L) | 0.941 | | **<0.001**** | | 0.839 | | **<0.001**** | |
| Blood uric acid (umol/L) | 0.927 | | **<0.001**** | | 0.941 | | **0.001**** | |
| NLRP3 total methylation level | 0.986 | | 0.531 | | 0.978 | | 0.207 | |
| AIM2 total methylation level | 0.851 | | **<0.001**** | | 0.892 | | **<0.001**** | |
| ASC total methylation level | 0.971 | | 0.126 | | 0.974 | | 0.123 | |

^*^ *P* < 0.05, ^**^ *P* < 0.001; The bold in the table means statistically significant. (*P* < 0.05)
